# Genetic Modification of Cytokine Signaling to Enhance Efficacy of CAR T Cell Therapy in Solid Tumors

**DOI:** 10.3389/fimmu.2021.738456

**Published:** 2021-10-14

**Authors:** Navid Ghahri-Saremi, Behnia Akbari, Tahereh Soltantoyeh, Jamshid Hadjati, Saba Ghassemi, Hamid Reza Mirzaei

**Affiliations:** ^1^ Department of Medical Immunology, School of Medicine, Tehran University of Medical Sciences, Tehran, Iran; ^2^ Center for Cellular Immunotherapies, Perelman School of Medicine, University of Pennsylvania, Philadelphia, PA, United States

**Keywords:** cytokines, chemokines, genetic modification, CAR T cell, immunotherapy, solid tumors

## Abstract

Chimeric antigen receptor (CAR) T cell therapy has shown unprecedented success in treating advanced hematological malignancies. Its effectiveness in solid tumors has been limited due to heterogeneous antigen expression, a suppressive tumor microenvironment, suboptimal trafficking to the tumor site and poor CAR T cell persistence. Several approaches have been developed to overcome these obstacles through various strategies including the genetic engineering of CAR T cells to blunt the signaling of immune inhibitory receptors as well as to modulate signaling of cytokine/chemokine molecules and their receptors. In this review we offer our perspective on how genetically modifying cytokine/chemokine molecules and their receptors can improve CAR T cell qualities such as functionality, persistence (e.g. resistance to pro-apoptotic signals) and infiltration into tumor sites. Understanding how such modifications can overcome barriers to CAR T cell effectiveness will undoubtedly enhance the potential of CAR T cells against solid tumors.

## Introduction

Surgery, chemotherapy and radiation therapy have been the principal cornerstones of cancer treatment since the middle of the last century. The development of novel molecular targeted therapies and immunotherapies such as immune checkpoint inhibitors and CAR T cells, among others, have led to a paradigm shift in the treatment of cancer patients (1, 2). Exciting progress with CD19-CAR T cells for the treatment of certain pediatric and young adult patients with B cell acute lymphoblastic leukemia has led to the successful approval by the US Food and Drug Administration (FDA) in August 2017 (3, 4). Despite the advancement in the treatment of blood cancers with CAR T cells, treating solid tumors has been challenging in part due to heterogeneous tumor antigen expression, the presence of immunosuppressive and hostile tumor microenvironment (TME) that exacerbates CAR T cell exhaustion and apoptosis, and insufficient infiltration into the tumor sites (5–7).

It is well-recognized that cytokines, chemokines and their receptors play pivotal roles in regulating the functional and phenotypic features of CAR T cells; influencing parameters such as persistence, trafficking, memory cell formation and proliferation. All of which are essential determinants of an effective therapy (8, 9). Cytokines and chemoattractant cytokines (also known as chemokines) are small glycoproteins that regulate immune cell activation, differentiation, growth and trafficking. Moreover, these small glycoproteins not only have critical roles in shaping of immune responses against different types of pathogens and tumor antigens but also determine which type of immune responses (e.g. cell-mediated *vs*. humoral immunity) should be developed to effectively eliminate their targets. Each cytokine has different functions and can provoke different responses depending on the target, cellular source and different phase of immune response. These small glycoproteins can have proinflammatory and anti-inflammatory properties which partly depend on the nature of target antigen and the context that an immune response initiated (10). Although systemic or local administration of cytokines in combination with CAR T cells improved antitumoral efficacy of these cells, some adverse effects including toxicity or even death, have been considered as significant barriers for systemic application of cytokines (11, 12).

Considering the importance of cytokines, chemokines and their receptors in the biology and immunology of CAR T cells, several investigators have tried to modulate the profile of cytokines and chemokines in CAR T cells or equip them with cytokines, chemokines or their receptors aiming to alleviate CAR T cell overactivation (i.e. cytokine release syndrome(CRS)) and/or overcome barriers (e.g. harsh immunosuppressive tumor microenvironment, suboptimal trafficking of CAR T cells to tumor site and activation-induced cell death) to their effectiveness. There are various strategies to genetically manipulate CAR T cells to enhance the overall functionality of these engineered cells against various types of tumors. For example, one of these strategies is the use of CRISPR-based gene editing technology to blunt the expression of cytokine genes responsible for CAR T cell dysfunction or CAR T cell-related toxicity (e.g. CRS). In this system, a single guide RNA (sgRNA) directs CAS9 endonuclease to the target gene. Binding of sgRNA to targeted gene activates CAS9 endonuclease which then leads to cleavage and knocking out of the target gene (13, 14). Another example is incorporation of truncated cytokine receptors into CAR T cells (through viral transduction) which lack a specific intracellular domain. These receptors block signal transduction of inhibitory cytokines and therefore enhance the persistence and effector function of CAR T cells (15, 16). Another approach is the co-expression of CAR construct with desirable cytokine through the 2A linker system or two vector systems. 2As are viral oligopeptides that can mediate cleavage of translated construct which leads to co-expression of both cytokine and CAR on T cells at the same time and expression level (17, 18). SynNotch receptor system and inducible promoter systems are examples of inducible expression of cytokines (19, 20). In the SynNotch receptor system, the recognition of specific antigen, mediates transcriptional activation of a specific cytokine gene in CAR T cells (21). Also, there are other strategies such as inverted cytokine receptors (ICRs) in which ectodomain of inhibitory cytokine is fused with endodomain of immunostimulatory cytokines (22), and membrane tethered cytokines that can be transferred *via* viral transduction or sleeping beauty (SB) system (excision and insertion of SB transposon into TA dinucleotide repeat of target-cell genome) (**Table 1**) (34, 46).In this review we discuss how employing various genetic strategies like incorporating dominant negative receptors, inverted cytokine receptors and immunostimulatory cytokines not only can diminish and/or reverse negative CAR T cell regulators in the tumor microenvironment but also can augment positive regulators of CAR T cells (e.g. proliferation and persistence) in solid tumors.

**Table 1:** Novel genetic modifications in cytokine, chemokine and their receptors to enhance the efficacy of CAR T cell therapy.

Strategies	Cytokines & Chemokines	Genetic modification	Reference
Improving persistence	IL-15 & IL-21	Overexpression	(23)
	IL-7 & CCL9	Overexpression	(24)
	IL-15	Overexpression	(25, 26)
	TGFβ	CRISPR-mediated knockout	(27)
	IL-7	ICR	(28)
Converting immuno-suppressive to immuno-promoting signals	IL-4R/IL-7R	ICR	(29)
	IL-4R/IL-21R	ICR	(22)
	TGFβ/IL-7R	ICR	(30)
	GM-CSF/IL-18R	ICR	(31)
Overcoming Exhaustion	TGFβRII	Truncated	(15)
		CRISPR-mediated knockout	(27)
	aPD1/TGFβ trap	Overexpression	(32)
	IL-18	Inducible expression	[20]
	IL-7	Overexpression	(33)
	IL-15	Membrane tethered	(34)
AICD prevention	IL-7	ICR	(28)
	IL-15	Membrane tethered	(34)
Improving infiltration	CCR6	Overexpression	(35)
	CCR2b	Overexpression	(18, 36)
	CCR4	Overexpression	(37)
	CXCR2	Overexpression	(38)
CRS and neurotoxicity inhibition	IL-6	shRNA-mediated Knockdown	(39)
	mbaIL-6	Truncated	(40)
	GM-CSF	CRISPR-mediated knockout	(41)
	GM-CSF & aIL-6/IL-1RA	CRISPR-mediated knockout & secreting neutralizing antibody & antagonist	(14)
Endogenous immune system activation	IL-7 & CCL19	Overexpression	(24)
	IL-18	Overexpression	(42)
	IL-12	Overexpression	(43, 44)
	IL-36γ	Overexpression	(45)

ICR: inverted cytokine receptor; CRS: cytokine release syndrome; mbaIL6: membrane-bound IL-6 receptor; aIL-6R: anti-IL-6 receptor antibody; IL-1RA: IL-1 receptor antagonist.

## Improving CAR T Cell Persistence

It is well-known that persistence of CAR T cells is directly correlated with durable clinical remissions in patients with cancers (47, 48). In fact, poor persistence potentially hinders the long-term therapeutic effects of CAR T cell *in vivo*. It has been shown that several parameters can affect the survival of adoptively transferred CAR T cells (49). In the two following sections, we discuss how genetic modification of CAR T cells to overexpress cytokines or their receptors makes prolong their survival.

### Production of Less Differentiated CAR T Cells

It is well-documented that the differentiation status of CAR T cells plays a prominent role in therapeutic success. It seems this successful therapeutic outcome is largely depend on the fact that less differentiated CAR T cells (e.g. naïve T cells (TN), stem cell memory (TSCM) and central memory (TCM)) are correlated with improved expansion, prolonged *in vivo* persistence, and long-term anti-tumor control (50).

As a result, many studies have focused on the production of CAR T cells with a less differentiated phenotype through employing different pharmacological and genetic mechanisms. For instance, less differentiated CAR T cells have been generated through inclusion of cytokine genes (e.g. IL-9, IL-7, IL-15 and IL-21) in the CAR gene construct (51, 52) and incorporation of cytokine-induced JAK/STAT signaling domains in the CAR gene construct (53–55). Using a hepatocellular carcinoma model, it has been revealed that co-incorporation of IL-15 and IL-21 genes into the anti-GPC3 CAR construct, leads to greater proliferation capacity, enhanced persistence and survival and elevated proportion of stem cell memory CAR T cell subpopulation (23). Adachi and colleagues also showed that IL-7 and CCL19 co-expressing CAR-T cells become differentiated into central memory CAR T cells with superior tumor-infiltrating capacity and higher persistence rate in a P815-hCD20 (mastocytoma) mouse model (24). Incorporation of IL-15 into CAR construct could also enhance stem-cell like memory CAR T cell portion with superior tumor killing ability and reduced expression of PD-1 receptor in neuroblastoma-bearing mice compared to conventional CAR T cells (25).

Using JAK/STAT signaling domains downstream of cytokine receptors is also another tactic for blunting CAR T cells differentiation towards terminally differentiated phenotype. The γc-family cytokine-stimulated JAK/STAT signaling pathway is shown to dampen CAR T cells phenotype into terminally-differentiated CAR T cells. Using a CAR construct encoding a truncated cytoplasmic domain from IL-2Rβ and a STAT3-binding tyrosine-X-X-glutamine (YXXQ) motif, together with the CD3z and CD28 domains [also referred to as 28-ΔIL2RB-z(YXXQ)], it has been exhibited that the 28-ΔIL2RB-z(YXXQ) CAR T cells are highly proliferative and are not vulnerable to the acquisition of terminally- differentiated phenotype in a B-ALL experimental model. Compared to CAR T cells without STAT3 motif, 28-ΔIL2RB-z(YXXQ) CAR T cells maintained proliferation, IL-2 secretion, cytokine polyfunctionality. These results suggest a key role of STAT3 in suppressing terminal differentiation of T cells, which is consistent with recent human and mouse studies (54, 55). Modified CAR T cells also expressed markers related to stem cell like memory phenotype (such as, CD27, CD28 and CD95) (53). These characteristics have been also described in less differentiated memory T cells (56, 57). Currently, a phase 1 clinical trial is being planned to investigate the effect of IL-15 and IL-21 armored Glypican-3-specific CAR T cells for pediatric solid tumors (NCT04715191).

### Production of CAR T Cells Resistant to Pro-Apoptotic Signaling Cues

Overexpression of proapoptotic proteins such as Bid, Bim and FasL has been related to progressive T cell differentiation and loss of self-renewal capacity (58). Resistance to pro-apoptotic signals and/or augmentation of anti-apoptotic signaling pathway are supposed to be an alternative option for promoting CAR T cell survival and persistence (49). TGF-β as a potent immunosuppressant of TME is produced by different cell types such as cancer associated fibroblasts, mesenchymal stem cells, lymphatic epithelial cells and blood endothelial cells (59). It is generally accepted that TGF-β inhibit T cell activation and proliferation likely due to induction of T cell apoptosis *via* either proapoptotic-dependent (e.g. BIM) or independent pathways (60–62). To blunt its suppressive effects on CAR T cells and to enhance the overall antitumor function of CAR T cells, CRISPR-mediated TGFβR2-knockout CART cells (TGFβR2.KO CART cells) have been developed. TGFβR2.KO CART cells displayed higher survival and proliferation rates and were more resistant to exhaustion in the pancreatic carcinoma-bearing mice (27). IL-15 is known as a general inhibitor of apoptosis, which possesses potential therapeutic properties. Overexpression of murine IL-15 in CAR T cells has led to generation of CAR T cells with enhanced persistence, lower level of PD-1 expression, being more resistant to proapoptotic signals (probably due to augmentation of BCL-2 level) and improved antitumor immune response in a B16 melanoma model *in vivo* compared to conventional 2nd generation CAR T cells (26). In addition, IL-7 signaling through STAT5 has shown to be in favor of CAR T cell resistance to proapoptotic signals. In a study, Shum and colleagues have shown that constitutive signaling downstream of IL-7 receptor, through using CD34 ectodomain and endodomain of IL-7Rα (C7R), leads to upregulation of anti-apoptotic protein BCL2 and downregulation of proapoptotic protein CASP8 (Caspase-8) in an orthotopic glioblastoma mouse model (28). Altogether, these findings indicate that genetic modification of cytokines and their receptors might make CAR T cells more resistant to negative regulators of persistence through reprogramming of CAR T cell differentiation and abrogation of proapoptotic signaling.

## Converting Immunosuppressive Signals to Immunopromoting Signals

Immunosuppressive cytokines including IL-10, TGFβ and IL-4, are one of the key components of TME contributing to CAR T cell dysfunction. These cytokines induce immunosuppression *via* several mechanisms such as recruitment and activation of regulatory T cells (Tregs), myeloid-derived suppressor cells (MDSCs), inhibition of the effector function of CAR T cells. In addition, they can inhibit the activity of several endogenous antitumor immune cells like T cells, NK cells, dendritic cells, and M1 macrophages. Immunosuppressive cytokines such as TGFβ can also disrupt the balance between TH1 and TH2 cells toward TH2 cells which leads to induction of other suppressive cytokines (e.g. IL-4) (63). To overcome cytokine-induced immunosuppression in the CAR T cells, Mohammed and colleagues have developed an inverted cytokine receptor IL-4R/IL-7R [i.e. 4/7 ICR, consisted of an IL-4R exodomain fused to an IL-7R endodomain]. Upon ligation to immunosuppressive cytokine IL-4, this chimeric switch receptor not only could significantly restrict the immunosuppressive effects of IL-4 but also could successfully convert inhibitory signals to IL-7 immunostimulatory signals. This immunostimulatory downstream signaling could prevent and/or restore CAR T cell exhaustion and dysfunction as well as could improve CAR T cell survival in a harsh TME of pancreatic cancer experimental model (29). In another study, Wang et al., have showed that overexpression of IL-4R/IL-21R inverted cytokine receptor (4/21 ICR) in CAR T cells could promote TH17-like polarization and enhance tumor specific cytotoxicity of 4/21 ICR-engineered CAR T cells in an IL-4-enriched hepatoma tumor milieu *via* activation of STAT3 pathway. Also, these cells were characterized by enhanced persistence and could successfully control established IL-4-secreting tumors *in vivo* (22). Weimin et al., have also reported that equipment of CAR T cells with chimeric cytokine switch receptor TGFβ/IL-7 not only could enhance their cytotoxic activity, cytokine production ability (e.g. IFNγ and TNFα) and proliferation capacity but also could reduce the expression of inhibitory receptors (e.g. PD-1 and LAG-3) in a prostate cancer model (30). Chimeric cytokine switch receptor GM-CSF/IL-18R (GM18) overexpressed in CAR T cells could also confer a higher rate of cellular expansion, cytokine production and sustain cytotoxic activity in a chronic antigen-stimulated condition of various preclinical EPHA2 or HER2 positive solid tumor models, compared to unmodified CAR T cells (31). In aggregate, it seems converting immunosuppressive cytokine signals to immunopromoting signals may be a promising strategy for improving CAR T cell functionality and longevity.

## Overcoming CAR T Cell Exhaustion

CAR T cell exhaustion represents a substantial barrier to the eradication of tumors and is associated with poor clinical outcome. Exhaustion is a common feature of tumor-infiltrating CAR T cells. Immunological exhaustion is characterized by progressive loss of T cell effector functions and proliferative capacity, sustained expression of inhibitory receptors (e.g. PD-1), increased susceptibility to apoptosis and activation of a transcriptional state distinct from that of functional effector or memory T cells (64). Tumor cells, and their surrounding immunosuppressive cells and cytokines are supposed to contribute to this exhausted T cell phenotype (65). As exhausted CAR T cells showed impaired effector function and failed to eradicate tumors (66), thus reversing this state can potentiate/restore the function of exhausted CAR T cells and thereby restore a robust antitumor response.

It has been shown that genetic modification of CAR T cells in this particular case-cytokine overexpression (e.g. IL-18, IL-15 and IL-7) or repression/abrogation (e.g. TGFβR) can be a powerful approach for reverting CAR T cell exhaustion. TGFβ is a well-known immunosuppressive cytokine. This cytokine can induce T cell exhaustion through downstream signaling molecules (e.g. SMAD2 and SMAD3) of its receptor (e.g. TGFβRII). Therefore, genetic modification of TGFβR signal transduction can be an efficient method for the prevention of CAR T cell exhaustion. One of the examples is generation of dominant negative TGFβRII CAR T cells which have truncated TGFβRII that lack an intracellular signaling domain. This method makes CAR T cells resistant to exhaustion, enhances their proliferation and cytokine production abilities and confers long-term persistence with effective antitumor response against PSMA positive prostate cancer cells (15). In another study using CRL5826 positive melanoma cells, Tang and colleagues have demonstrated that knocking out the endogenous TGFβRII in CAR T cells with CRISPR/Cas9 technology could reduce the induced Treg conversion and prevent the exhaustion of CAR T cells (27). Secretion of bispecific protein of anti-PD-1 fused with TGF-β trap has also shown to enhances antitumor efficacy of CAR T cells through attenuating inhibitory T cell signaling, enhancing T cell persistence and expansion, and improving effector function and resistance to exhaustion in a prostate cancer xenograft mouse model (32). It has been well-documented that overexpression of cytokines (e.g. IL-18, IL-7 and IL-15) can prevent/revert CAR T cell exhaustion. Chmielewski and colleagues have revealed that CAR T cells engineered with inducible IL-18 release, as a potent immune modifier, can prevent CAR T cell exhaustion in large pancreatic and lung tumor models (20). Previous studies have also suggested an anti-exhaustive role for IL-7 (67). IL-7 secreting CAR T cells were also shown to express lower levels of exhaustion markers (e.g. PD-1 and LAG3) and higher levels of anti- transcription factor TCF-1, a transcription factor that is supposed to counteract exhaustion programs, in a gastric cancer experimental model (33, 68, 69). IL-15 has also proven beneficial in antagonizing CAR T cell exhaustion (70, 71). In agreement with these reports, Singh and colleagues have demonstrated that CAR T cells expressing a membrane-bound chimeric IL-15 (mbIL15) are not only characterized by long-term persistence with a memory stem-cell phenotype but also express lower levels of exhaustion markers and higher expression level of anti-exhaustive transcription factor TCF-1 in a xenograft mouse model of leukemia (34). Furthermore, Narayan et al. have conducted a phase 1 clinical trial for dominant negative TGFβR CAR T cell (PSMA-directed/TGFβ-insensitive CAR T cells) against metastatic castration-resistant prostate cancer (CRPC). Cohorts 1 and 2 have been done without observed dose-limiting toxicity (DLT). Intriguingly, a cytokine release syndrome has been observed that is reversible and responsive to tocilizumab (72).

## Overcoming Activation-Induced Cell Death (AICD)

Activation‐induced cell death (AICD) is a major mechanism of T cell homeostasis and acts to prevent excessive T cell responses towards possible subsequential autoimmunity (73). Induced by repeated antigen stimulation under particular conditions, T cells undergo apoptosis in a controlled manner through the engagement of death receptors (e.g. Fas) and activation of specific caspases (e.g. caspase-8). Although AICD is generally considered as a T cell regulatory mechanism in the physiological conditions, this process is also triggered in the TME following chronic activation of tumor-infiltrating T cells (e.g. adoptively transferred CAR T cells), leading to apoptosis of tumor-redirected CAR T lymphocytes, thereby, hampering their full therapeutic potential (74).

Several efforts have been made over the years to overcome this barrier aiming to preserve CAR T cell efficacy and improve their survival and persistence in the TME. It has been shown that various cytokines are involved in either induction (e.g. TNFα and IL-2) or prevention (e.g. IL-7 and IL-15) of AICD. As mentioned above, Shum et al., have exhibited that CAR T cells supporting constitutive signaling downstream of IL-7 receptor have lower levels of Fas and proapoptotic protein CASP8 (Caspase-8), two major proteins that are involved in AICD (28). These findings highlight the role of IL-7 and its related signaling pathways in the inhibition of AICD. IL-15 is another potential cytokine for antagonizing AICD. The role of IL-15 in preventing AICD has been very well established (75). For instance, it has been shown that engineering of CAR T cells with membraned-bound IL-15 makes them more resistant to AICD (34). In other hand, it has been reported that cytokines like IL-2, IL-4, TNFα and IFNγ are in favor of AICD induction (76). Therefore, it seems that genetic abrogation of these cytokines may alleviate the role of these cytokines in the induction of AICD in CAR T cells, however, it is remained to be further studied in future (76). In conclusion, these findings indicate that genetic modification and/or targeting of specific cytokine genes and/or their receptors may be a good option for overcoming activation-induced cell death in the CAR T cells, thereby, improving the efficacy of CAR T cell therapy.

## Improving CAR T Cell Infiltration Into Tumor Site

Suboptimal trafficking of CAR T cells to the tumor sites represents another hurdle to CAR T cell therapy. Several reports have demonstrated that enhanced trafficking of adoptively transferred-CAR T cells to tumor sites is correlated to their therapeutic efficacy and clinical outcome in the cancer patients (77–79).

Various barriers that hinder optimal trafficking of CAR T cells to the tumor sites have been described. These barriers include: i) chemokine/receptor mismatch between the CAR T cell chemokine receptors and the chemokines secreted by tumors (e.g. such as CXCL1, CXCL5 and CXCL12), ii) low levels of tumor-derived chemokines for which effector CAR T cells lack receptors, iii) abnormal tumor vascularity and iv) physical (e.g. extracellular matrix (ECM]) and cellular barriers (e.g. cancer associated-fibroblasts [CAFs]) (80, 81). Although various strategies such as local delivery of CAR T cells, targeting tumor-related cellular and physical barriers (e.g. generation of anti-FAP CAR T cells targeting CAFs and CAR T cells to overexpress heparanase, an ECM-degrading enzyme) and targeting abnormal tumor vascularity (e.g. generation of CAR T cells targeting VEGFR2 expressed tumor‐associated blood vessels) have been employed to overcome CAR T cell infiltration, genetic modification of chemokine receptors, among the others, has been the most common strategy to improve CAR T cell infiltration into the tumor bed (82–84). Genetic modifications of these molecules have widely been used as a novel strategy for conferring new migratory capacity to administrated CAR T cells. Jin et al. showed that anti-EGFR CAR T cell migration to lung cancer site was enhanced by overexpression of CCR6, which recognizes lung cancer-produced CCL20, a chemokine that is highly expressed by lung adenocarcinoma cells. The authors also found that overexpression of CCR6 has no negative effects on the CAR T cell effector functions and their phenotype. In addition, mice receiving CCR6-overexpressing CAR T cells showed enhanced survival and an improved antitumor activity compared to mice receiving conventional unmodified CAR T cells (35). AS chemokine CCL20 is also overexpressed in various types of cancer like colon adenocarcinoma (COAD), rectum adenocarcinoma (READ) and stomach adenocarcinoma (STAD), therefore, overexpression of CCR6 in CAR T cells might be an effective strategy to overcome insufficient infiltration of CAR T cells into CCL20-expressing tumor sites. CCR2b is a chemokine receptor that poorly expressed in all resting and activated peripheral blood T cells and IL-2 activated malignant pleural mesothelioma (MPM)-infiltrating lymphocytes. To promote infiltration of CAR T cells into MPM-bearing sites, Moon et al., generated an anti-mesoCAR T cells overexpressing CCR2b (i.e. CCR2b-mesoCAR T cells). Their data showed that overexpression of CCR2b in mesoCAR T cells can significantly increase their migration to mesothelin+ MPM sites *in vivo*, leading to enhanced antitumor effects (18). A separate report showed that expression of CCR2b on GD2-CAR T cells significantly increase migration of CAR T cells toward CCL2 secreting neuroblastoma cells (36). Di Stasi et al., have reported that overexpression of CCR4 on anti-CD30 CAR T cells improved the trafficking of these engineered T cells toward CCL17-Hodgkin lymphoma cells (37). Another report also proved that overexpression of CXCR2 on CAR T cells increase homing capability of CXCR2-expressing CAR T cells toward hepatocellular carcinoma cells-producing CXCL1, CXCL2, CXCL3, CXCL5, CXCL6, and CXCL8 (38). In aggregate, these data indicate that genetic modification of chemokine receptors in CAR T cells may be a novel strategy to improve the efficacy and homing capabilities of adoptively transferred CAR T cells.

## Modulating Cytokine Release Syndrome and Neurotoxicity Following CAR T Cell Therapy

Systemic cytokine release [also known as cytokine release syndrome (CRS)] is a common but potentially fetal adverse event following CAR T cell therapy (85). Following the administration of CAR T cells, an exaggerated systemic immune response mediated by activated CAR T cells and various endogenous immune system components (e.g. monocytes/macrophages) is initiated. This acute systemic inflammatory response is mainly triggered by the release of a large amount of inflammatory mediators such as cytokines (e.g. IL-6, IL-1, IFN-g and GM-CSF) and chemokines [e.g. MCP-1 and MIP-1α] (86). This acute inflammatory response also induce endothelial and organ injury, which leads to microvascular leakage, heart failure and even death (85). Therefore, timely and properly interventional strategies that control CRS symptoms better or even to prevent CRS and neurotoxicity associated with CAR T cell therapy while preserving the efficacy CAR T cell treatment is of great importance and are urgently needed. Unlike hematological malignancies, the data on CRS incidence in solid tumors is limited probably due to the existence of immunosuppressive TME and insufficient infiltration of CAR T cells to tumor site (87–90). Thus, in this section will discuss different strategies that have been employed to overcome CRS in both hematological and non-hematological cancers.

Since it has been shown that IL-6 is the key molecule of CRS, many studies have recently focused to reduce and/or overcome IL-6-mediated CRS (91, 92). To do so, two independent studies have knocked down IL-6 gene in anti-CD19 CAR T cells *via* incorporation of short hairpin RNA (shRNA) into CAR construct (termed ssCART-19) (39, 92). Their data revealed that IL-6 released from CAR T cells not only cause CRS but also induces secretion of proinflammatory cytokines in the monocytes which altogether participate in the incidence and exacerbation of CRS. The authors also found that ssCART-19 cells produce lower levels of IL-6 and significantly reduce IL-6 secretion by monocytes in xenograft mouse model of leukemia. No significant difference in CAR T cell functionality in terms of proliferation and cytotoxicity was observed in both ssCART-19 cells and regular CART-19 cells. Reduced production of IL-6 by both CAR T cells and monocytes might lead to a significant reduction in the CRS incidence in the patients receiving CAR T cell therapy (39, 92).

In another study, Tan et al., have engineered anti-CD19 CAR T cells to express a nonsignaling membrane bound IL-6 receptor (mbaIL6). *In vitro* testing of mbaIL6-expressing CAR T cells (termed mbaIL6CAR T cells) revealed similar cytotoxic and proliferative abilities compared to conventional CAR T cells. Moreover, mbaIL6CAR T cells were able to neutralize macrophage-derived IL-6 while preserving their powerful antitumor activity *in vitro*. *In vivo* studies using CD19+ ALL cell line Nalm-6 also showed that anti-CD19CAR T cells were effective in targeting of CD19+tumor cells regardless of mbaIL6 expression. However, level of human IL-6 in mice was significantly diminished in the mbaIL6CAR T-treated tumor-bearing mice compared to unmodified anti-19CAR T cells (40). GM-CSF has been also identified as a crucial cytokine in the development of neurotoxicity and CRS. Elevated levels of GM-CSF promote secretion of IL-6, IL-8 and MCP-1, as other important CRS biomarkers, from monocytes (41). To prevent or reduce the risk of CRS and neuroinflammation mediated by GM-CSF following CAR T cell therapy, Sterner and colleagues have utilized lenzilumab, a GM-CSF neutralizing antibody, in combination with anti-CD19CAR T cells. Their data showed that blocking GM-CSF leads to enhanced CART cells proliferation and efficacy. They found that blocking of GM-CSF has no significant effect on the tumor killing capacity of CAR T cells in the presence of monocytes *in vitro.* In line with their *in vitro* findings, *in vivo* studies also revealed that lenzilumab not only inhibits GM-CSF effector functions, but also preserves anti-leukemia activity. *In vivo* studies also proved that CAR T 19 cells in combination with GM-CSF neutralizing antibody could significantly reduce neuroinflammation and prevent of CRS. To rule out any critical role for GM-CSF in CAR T cell function, the authors also generated GM-CSF knockout anti-CD19 CAR T cells (termed GM-CSFK.O CAR T 19 cells) using CRISPR/Cas9 gene editing technology. Their findings revealed that GM-CSFK.O CART19 cells could significantly reduce GM-CSF production compared to conventional CART19 cells. In addition, gene-editing strategy had no interventional effect on the production of other effector cytokines (e.g. IFNγ and IL-2) in the gene-edited anti-CD19 CAR T cells *in vitro*. While GM-CSFK.O CAR T19 cells were not able to produce GM-CSF, they could control leukemia growth *in vivo* (41). Another example is production of CRISPR-edited GM-CSF knockout CAR T cells secreting anti-IL-6 scFv and IL1RA with TCR knockout (CART-aIL6/IL1RA with GM-CSF/TCR KO). Compared to GM-CSF wild type counterparts, CART-aIL6/IL1RA with GM-CSF/TCR KO showed similar cytotoxicity and reduced GM-CSF production against CD19+ Nalm6 leukemia cells. Also, a pilot study of three patients, one with refractory Non-Hodgkin lymphoma (NHL) and two with multiple myelomas (MMs) with CART-aIL6/IL1RA with GM-CSF/TCR KO showed 3/3 complete response, 2/3 with no CRS incidence, one with grade 2 CRS incidence and no neurotoxicity which proved safety and efficacy of CART-aIL6/IL1RA with GM-CSF/TCR KO. In addition, cytokine analysis revealed a low level of GM-CSF, IL-6 and IL-1β and elevated level of IL1RA in the treated patients (14). It was also reported that genetic disruption of GM-CSF in the CAR T cells can abolish macrophage-dependent secretion of CRS mediators, including Il-6, IL-8 and MCP-1 (93). Furthermore, Kang et al. have conducted a clinical trial to evaluate safety and efficacy of ssCART-19 in patients with acute lymphoblastic leukemia (CLL). Their data exhibited a significant reduction of severe CRS incidence in patients receiving ssCART-19 compared to those who received regular CART-19 (39). Neurotoxicity, also referred to as immune effector cell-associated neurotoxicity syndrome (ICANS), is another CAR T cell-related toxicity which often occurs and correlates with CRS, but it has also been sometimes reported to occur independently from CRS. The data obtained from preclinical studies revealed that monocyte-derived IL-1 appeared to mediate neurotoxicity and CRS (94). Stimulation of monocytes by GM-CSF following CART cell therapy was shown to be related to neuroinflammation in tumor-bearing mice (41). High levels of IL-6 and GM-CSF was detected in the cerebrospinal fluid (CSF) of non-human primate models of neurotoxicity following CART cell therapy (95). After CAR T cell therapy, cytokines, especially IL-1 and IL-6 and GM-CSF, have been demonstrated to promote systemic inflammation which associates with the development of severe neurotoxicity (96). GM-CSF has been also described as the cytokine most significantly correlated to the development of neurotoxicity following CART cell therapy in the ZUMA-1 clinical trial (97). These findings not only describe the crucial role of cytokines in pathogenesis of neurotoxicity but also highlight how genetic modification of cytokines by various strategies like shRNA-mediated knocking down of cytokine genes, design of nonsignaling membrane bound cytokine receptors and knocking out of cytokine genes in CAR T cells may significantly prevent and/or alleviate the post-CAR T cell therapy-related neurotoxicity.

In the case of solid tumors, although rare, but CRS incidence can occur much like happen in B-NHL as characterized by local CRS (L-CRS or compartmental-CRS) followed by systemic CRS (S-CRS). It seems immunosuppressive TME and suboptimal trafficking of CAR T cells to tumor bed prevent optimal tumor antigen recognition and therefore limit full activation of CAR T cells and subsequent cytokine release and CRS incidence (87). There are a few reports on the incidence of compartmental CRS in a patient with recurrent ovarian cancer after treatment with anti-mesothelin CAR T cell (98) or the occurrence of severe CRS in a 45-year-old patient with malignant mesothelioma after the treatment with anti-EpCAM CAR T cells (87). Altogether, these data suggest that genetic modification of cytokines and receptors in CAR T cells would be an appealing strategy to prevent CRS and neurotoxicity or reduce its severity without affecting the antitumor potential of CAR T cell therapy.

## Activation of Endogenous Immune System

**Figure 1:**
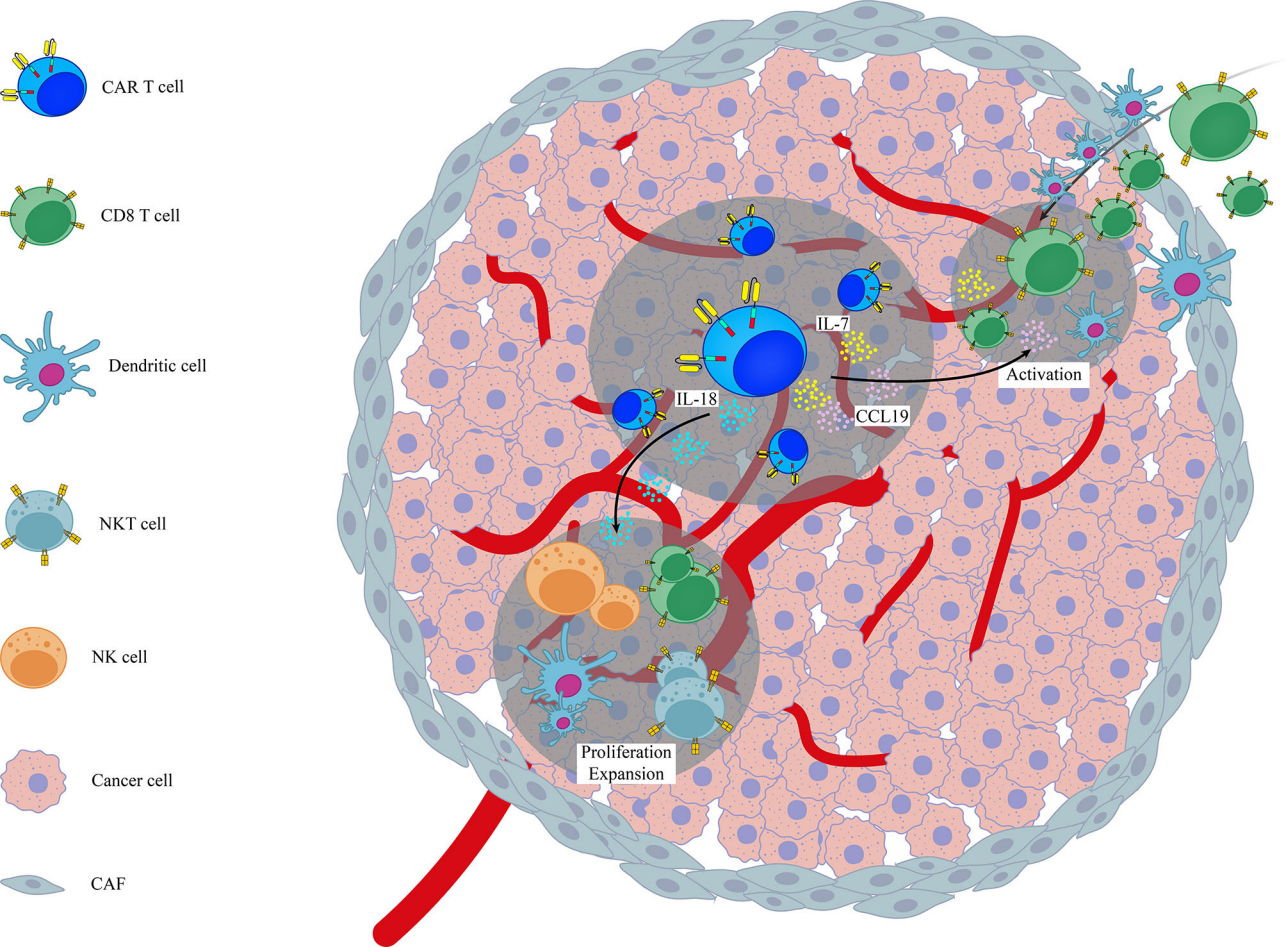
A schematic representation on the role of genetic modification of different cytokines/chemokines in activation of endogenous immune system in the context of CAR T cell therapy. Examples are based on the role of genetically modified CAR T cells (7x19 CAR T cells and IL-18 armored CAR T cells) in the activation of endogenous immune cells like CD8+ T cells, NK, NKT and DCs. CAF, Cancer Associated Fibroblasts.

Although genetically-modified CAR T cells have shown promising results compared to conventional CAR T cells, yet converting the CAR T cell response to a stronger and more continual one remains to be an important issue. CAR T cells are programmed to recognize one to three specific antigens utmost, but due to the pressure of immune selection and tumor antigen heterogeneity, some antigen-negative tumor variants can outgrowth and outperform the a successful antitumor function of administrated CAR T cells. One overcoming solution would be the induction of epitope spreading towards antigens beyond those recognized by adoptively transferred CAR T cells. Epitope spreading is characterized by the enhancement and diversification of the endogenous T-cell-mediated immune response against non-CAR antigenic epitopes (99, 100). Based on this strategy, stimulation of endogenous immune cells along with CAR T cells can collaboratively target tumor cells (**Figure 1**). It seems that the inflammatory environment made by engineered CAR T cells can result in priming of endogenous immune cells against additional target antigens that is beyond the CAR target and are present on tumor cells.

Various studies have shown that infiltration of endogenous immune cells (in particular T lymphocytes) into the tumor sites can expand the efficacy of CAR T cell therapy (87, 101). In line with this notion, Adachi et al., reported that overexpression of IL-7 and CCL19, as two essential cytokines for generation of less-differentiated, long-lived non-exhausted (CAR) T cells and recruitment of endogenous DCs and T cells, in CAR T cells (termed 7x19 CAR T cells) increased infiltration of DCs and T cells into tumor sites following 7 × 19 CAR T cell therapy. They also found that depletion of recipient T cells before 7 × 19 CAR T cell therapy diminished the therapeutic efficacy of these genetically-modified CAR T cells, indicating that CAR T cells and endogenous immune cells collaboratively exert antitumor activity (24). Avanzi and colleagues also demonstrated that IL-18 armored CAR T cells, unlike unmodified CAR T cells, were able to activate and recruit endogenous antitumor immune effector cells such as CD8 T cells, DCs, NK cells and NKT cells into B-ALL tumor sites and metastatic ovarian tumor sites broadening the antitumor response beyond the CAR target (42). Arming CAR T cells with IL-12, a potent immunostimulatory cytokine that activates the innate and adaptive cellular immune system, was also shown to enhance antitumor efficacy through TME reprogramming and activation of endogenous immune system with antitumor function in both lymphoma and ovarian tumor models (43, 44). However, it should be noted that in study conducted by the Koneru et al, the total concentration of serum IL-12 was consisted of both endogenous and exogenous IL-12, and was not a reflection of IL-12 solely produced by IL-12 armored-CAR T cells (102). In another independent study, Kueberuwa et al. have exhibited that IL-12-expressing anti-CD19CAR T cells not only directly kill lymphoma cells, but also recruit host antitumor immune effector cells to an anti-cancer immune response in the lymphoreplete mice (103). CAR T cells secreting IL-36γ have also shown higher expansion rate with superior antitumor function compared with unmodified CAR T cells. Their data also revealed that IL-36γ armored CAR T cells activate endogenous antigen-presenting cells (APCs) and T cells through promotion of a secondary antitumor response and delayed the progression of antigen-negative tumor challenge in an experimental model of B-cell lymphoma (45). A separate study has also reported that effective CAR T cell antitumor activity of IL13Ra2-CAR T cells against mouse syngeneic glioblastoma (GBM) is significantly dependent on the activation of patient-derived endogenous T cells and monocyte/macrophages at the tumor site in an IFNγ-dependent manner (101). Currently, a phase 2 clinical trial for IL-7 and CCL-19 expressing CAR T cells against Refractory/Relapsed B Cell Lymphoma is in recruiting status (NCT03929107). Altogether, these findings highlight the importance of activation of endogenous immune system in the context of CAR T cell therapy. It seems CAR T cells act as immunomodulatory adjuvant for the activation of host immune cells. This insight strongly supports the need to consider targeting/engaging host immunity to improve the efficacy of CAR T cell therapy. Moreover, activation of endogenous immune system can additionally prevent or delay the progression of antigen–negative tumor variants.

## Concluding Remarks

**Figure 2:**
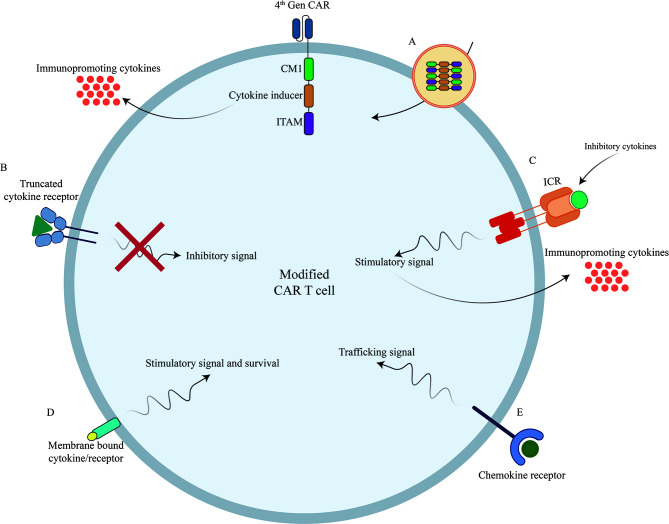
Novel strategies to boost CAR T cells efficacy against different types of tumors using genetic manipulations of different cytokines/chemokines and their receptors. **(A)** Incorporation of different cytokines/chemokines or their signaling domains in CAR construct. **(B)** Inclusion of truncated cytokine receptors lacking intracellular signaling domain. **(C)** Incorporation of inverted cytokine receptors (ICRs) to convert immunosuppressive signals to immunopromoting signals **(D)** Incorporation of membrane-tethered cytokine to provoke certain signal transduction of through its receptor. **(E)** Overexpression of chemokine receptors to improve CAR T cell trafficking into the tumor sites. CAR, Chimeric Antigen Receptor; TCR, T Cell Receptor; ICR, Inverted Cytokine Receptor.

Although CAR T cell therapy has made great strides in the treatment of patients with advanced blood cancers; their success in solid tumors has been limited partly due to the cellular, molecular and physical barriers of the TME. Developing innovative approaches including arming CARTs with cytokine signaling modalities to overcome these barriers has important translational relevance. In this review, we outline several strategies to enhance the effectiveness of CAR T cells emphasizing roles for several cytokines, chemokines and their signaling pathways to overcome and/or prevent CAR T cell dysfunction or hyperactivation (**Figure 2**).

Genetically modifying cytokine/chemokine signaling pathways is an appealing approach to enhance the therapeutic properties of CAR T cell therapy for solid tumors. It is anticipated that new generations of cytokine/chemokine-gene-modified CAR T cells could effectively target/engage endogenous immune system to synergistically improve overall antitumor immunity and additionally prevent the appearance of antigen-negative tumor variants, and thereby, tumor relapse in the context of CAR T cell therapy. However, CRS and ICANS incidences remain main safety concerns as CRS and ICANS are potentially fatal adverse effect of CAR T cell therapy and genetic modifications of cytokines/chemokines that enhance CAR T cell function could exacerbate CRS and ICANS. To date, nearly all of studies have only investigated constitutive expression of single chemokine/cytokine. Thus, generation of CAR T cells engineered with an inducible chemokine/cytokine platform not only can increase efficacy but also guarantee safety of CAR T cell therapy.

Due to the fact that cytokine/chemokine gene expression profile can be various between different individuals or even at different stages of same tumor in an individual, hence; analysis of cytokine/chemokine gene expression signature in TME before CAR T cell therapy could aid scientists to individualize cytokine engineered-based CAR T cell therapy. Although genetic modification of cytokine signaling in CAR T cells have shown promising clinical results in both hematologic and non-hematologic cancers (**Table 2**), it seems combination of different genetic modification approaches could be beneficial as some of cytokines are immuno-inhibitory and others are immuno-stimulatory. Thus, modification of immunoinhibitory pathways using gene editing technologies (e.g. CRISPR/Cas9) or use of truncated cytokine receptors or cytokine traps alongside with overexpression of immune-stimulatory cytokines may reprogram TME and significantly improve the efficacy of CAR T cell therapy.

**Table 2:** Selected clinical trials with cytokine/chemokine genetically-modified CAR T cells.

Clinical trial ID	Status	Cytokine/Chemokine Genetic Modification	CAR	Condition or disease	Phase
NCT04099797	Recruiting	C7R^1^	GD2-CAR	High Grade Glioma/Diffuse Intrinsic Pontine Glioma/Medulloblastoma	Phase 1
NCT03635632	Recruiting	C7R	GD2-CAR	Relapsed/Refractory Neuroblastoma	Phase 1
NCT03198546	Recruiting	IL-7 and CCL19	GPC3-CAR	Hepatocellular Carcinoma or Squamous Cell Lung Cancer	Phase 1
NCT03929107	Recruiting	IL-7 and CCL19	CD19-CAR	Refractory/Relapsed B Cell Lymphoma	Phase 2
NCT04381741	Recruiting	IL-7 and CCL19	CD19-CAR	Relapsed/Refractory Diffuse Large B Cell Lymphoma	Phase 1
NCT03778346	Recruiting	IL-7 and CCL19	BCMA-CAR, CD138-CAR, CD38-CAR, Integrin β7-CAR, CS1-CAR	Relapsed/Refractory Multiple Myeloma	Phase 1
NCT03932565	Recruiting	IL-7 and CCL19, or IL-12	Nectin4/FAP-CAR	Solid Tumors	Phase 1
NCT03721068	Recruiting	IL-15	GD2-CAR	Relapsed/Refractory Neuroblastoma or Relapsed/Refractory Osteosarcoma	Phase 1
NCT04377932	Recruiting	IL-15	GPC3-CAR	Pediatric Solid Tumors	Phase 1
NCT03579888	Terminated	mbIL-15^2^	CD19-CAR	B Cell Lymphoma and Leukemia	Phase 1
NCT04715191	Not yet recruiting	IL-15 and IL-21	GPC3-CAR	Pediatric Solid Tumors	Phase 1
NCT02498912	Active, not recruiting	IL-12	4H11-CAR	MUC16ecto^+^ Solid Tumors	Phase 1
NCT03542799	Unknown	IL-12	EGFR-CAR	Metastatic Colorectal Cancer	Phase 1 and 2
NCT01818323	Recruiting	IL-4Rα and IL-2Rβ (4αβ)	T1E-CAR	Head and Neck Squamous Cell Carcinoma	Phase 1
NCT04153799	Recruiting	CXCR5	EGFR-CAR	Non-Small Cell Lung Cancer	Phase 1

Finally, it seems that a more comprehensive understanding of the relevant cellular and molecular adaptations to tumor cells and immunological processes in their surrounding microenvironment will help us develop new generations of cytokine/chemokine-gene-modified CAR T cells that are more potent in overcoming the challenges of solid tumors.

## Author Contributions

NG-S, SG, and HM conceived the idea of the manuscript. NG-S, BA, and TS performed the bibliographic research, wrote the original draft and drew the figures. HM, SG, and JH edited and critically revised the manuscript. All authors contributed to the article and approved the submitted version.

## Funding

This work was supported in part through funding provided by Tehran University of Medical Sciences (grants numbers 50756 and 50760, awarded to HM), St. Baldrick’s Foundation Scholar Award (SG), National Blood Foundation Scientific Research Grant Award (SG), as well as Office of the Assistant Secretary of Defense for Health Affairs through the Peer Reviewed Cancer Research Program under Award No. W81XWH-20-1-0417 (SG).

## Conflict of Interest

The authors declare that the research was conducted in the absence of any commercial or financial relationships that could be construed as a potential conflict of interest.

## Publisher’s Note

All claims expressed in this article are solely those of the authors and do not necessarily represent those of their affiliated organizations, or those of the publisher, the editors and the reviewers. Any product that may be evaluated in this article, or claim that may be made by its manufacturer, is not guaranteed or endorsed by the publisher.

## References

[1] Miliotou AN Papadopoulou LC. CAR T-Cell Therapy: A New Era in Cancer Immunotherapy. Curr Pharm Biotechnol (2018) 19:5–18. doi: 10.2174/1389201019666180418095526 29667553

[2] Ramalingam SS Owonikoko TK Khuri FR. Lung Cancer: New Biological Insights and Recent Therapeutic Advances. CA: Cancer J Clin (2011) 61:91–112. doi: 10.3322/caac.20102 21303969

[3] Cummins KD Gill S. Anti-CD123 Chimeric Antigen Receptor T-Cells (CART): An Evolving Treatment Strategy for Hematological Malignancies, and a Potential Ace-in-the-Hole Against Antigen-Negative Relapse. Leuk Lymphoma (2018) 59:1539–53. doi: 10.1080/10428194.2017.1375107 28901790

[4] Brudno JN Kochenderfer JN. Recent Advances in CAR T-Cell Toxicity: Mechanisms, Manifestations and Management. Blood Rev (2019) 34:45–55. doi: 10.1016/j.blre.2018.11.002 30528964 PMC6628697

[5] Mirzaei HR Rodriguez A Shepphird J Brown CE Badie B. Chimeric Antigen Receptors T Cell Therapy in Solid Tumor: Challenges and Clinical Applications. Front Immunol (2017) 8:1850. doi: 10.3389/fimmu.2017.01850 29312333 PMC5744011

[6] Rodriguez-Garcia A Palazon A Noguera-Ortega E Powell DJ Jr. Guedan S. CAR-T Cells Hit the Tumor Microenvironment: Strategies to Overcome Tumor Escape. Front Immunol (2020) 11:1109. doi: 10.3389/fimmu.2020.01109 32625204 PMC7311654

[7] Slaney CY Kershaw MH Darcy PK. Trafficking of T Cells Into Tumors. Cancer Res (2014) 74:7168–74. doi: 10.1158/0008-5472.CAN-14-2458 25477332

[8] Jin J Cheng J Huang M Luo H Zhou J. Fueling Chimeric Antigen Receptor T Cells With Cytokines. Am J Cancer Res (2020) 10:4038–55. PMC7783740 33414984

[9] Zhou J Jin L Wang F Zhang Y Liu B Zhao T. Chimeric Antigen Receptor T (CAR-T) Cells Expanded With IL-7/IL-15 Mediate Superior Antitumor Effects. Protein Cell (2019) 10:764–9. doi: 10.1007/s13238-019-0643-y PMC6776495 31250350

[10] Borish LC Steinke JW. 2. Cytokines and Chemokines. J Allergy Clin Immunol (2003) 111:S460–475. doi: 10.1067/mai.2003.108 12592293

[11] Yeku OO Purdon TJ Koneru M Spriggs D Brentjens RJ. Armored CAR T Cells Enhance Antitumor Efficacy and Overcome the Tumor Microenvironment. Sci Rep (2017) 7:10541. doi: 10.1038/s41598-017-10940-8 28874817 PMC5585170

[12] Hawkins ER D'Souza RR Klampatsa A. Armored CAR T-Cells: The Next Chapter in T-Cell Cancer Immunotherapy. Biol Targets Ther (2021) 15:95–105. doi: 10.2147/BTT.S291768 PMC8053711 33883875

[13] Bak RO Gomez-Ospina N Porteus MH. Gene Editing on Center Stage. Trends Genet TIG (2018) 34:600–11. doi: 10.1016/j.tig.2018.05.004 29908711

[14] Yi Y Chai X Zheng L Zhang Y Shen J Hu B. CRISPR-Edited CART With GM-CSF Knockout and Auto Secretion of IL6 and IL1 Blockers in Patients With Hematologic Malignancy. Cell Discovery (2021) 7:27. doi: 10.1038/s41421-021-00255-4 33907185 PMC8079381

[15] Kloss CC Lee J Zhang A Chen F Melenhorst JJ Lacey SF. Dominant-Negative TGF-β Receptor Enhances PSMA-Targeted Human CAR T Cell Proliferation And Augments Prostate Cancer Eradication. Mol Ther (2018) 26:1855–66. doi: 10.1016/j.ymthe.2018.05.003 PMC6037129 29807781

[16] Wieser R Attisano L Wrana JL Massagué J. Signaling Activity of Transforming Growth Factor Beta Type II Receptors Lacking Specific Domains in the Cytoplasmic Region. Mol Cell Biol (1993) 13:7239–47. doi: 10.1128/MCB.13.12.7239 PMC364794 8246946

[17] Liu Z Chen O Wall JBJ Zheng M Zhou Y Wang L. Systematic Comparison of 2A Peptides for Cloning Multi-Genes in a Polycistronic Vector. Sci Rep (2017) 7:2193. doi: 10.1038/s41598-017-02460-2 28526819 PMC5438344

[18] Moon EK Carpenito C Sun J Wang LC Kapoor V Predina J. Expression of a Functional CCR2 Receptor Enhances Tumor Localization and Tumor Eradication by Retargeted Human T Cells Expressing a Mesothelin-Specific Chimeric Antibody Receptor. Clin Cancer Res (2011) 17:4719–30. doi: 10.1158/1078-0432.CCR-11-0351 PMC3612507 21610146

[19] Luo H Wu X Sun R Su J Wang Y Dong Y. Target-Dependent Expression of IL12 by Synnotch Receptor-Engineered NK92 Cells Increases the Antitumor Activities of CAR-T Cells. Front Oncol (2019) 9:1448. doi: 10.3389/fonc.2019.01448 31921693 PMC6930917

[20] Chmielewski M Abken H. CAR T Cells Releasing IL-18 Convert to T-Bet(high) FoxO1(low) Effectors That Exhibit Augmented Activity Against Advanced Solid Tumors. Cell Rep (2017) 21:3205–19. doi: 10.1016/j.celrep.2017.11.063 29241547

[21] Roybal KT Williams JZ Morsut L Rupp LJ Kolinko I Choe JH. Engineering T Cells With Customized Therapeutic Response Programs Using Synthetic Notch Receptors. Cell (2016) 167:419–32.e416. doi: 10.1016/j.cell.2016.09.011 27693353 PMC5072533

[22] Wang Y Jiang H Luo H Sun Y Shi B Sun R. An IL-4/21 Inverted Cytokine Receptor Improving CAR-T Cell Potency in Immunosuppressive Solid-Tumor Microenvironment. Front Immunol (2019) 10:1691. doi: 10.3389/fimmu.2019.01691 31379876 PMC6658891

[23] Batra SA Rathi P Guo L Courtney AN Fleurence J Balzeau J. Glypican-3-Specific CAR T Cells Coexpressing IL15 and IL21 Have Superior Expansion and Antitumor Activity Against Hepatocellular Carcinoma. Cancer Immunol Res (2020) 8:309–20. doi: 10.1158/2326-6066.CIR-19-0293 PMC10765595 31953246

[24] Adachi K Kano Y Nagai T Okuyama N Sakoda Y Tamada K. IL-7 and CCL19 Expression in CAR-T Cells Improves Immune Cell Infiltration and CAR-T Cell Survival in the Tumor. Nat Biotechnol (2018) 36:346–51. doi: 10.1038/nbt.4086 29505028

[25] Chen Y Sun C Landoni E Metelitsa L Dotti G Savoldo B. Eradication of Neuroblastoma by T Cells Redirected With an Optimized GD2-Specific Chimeric Antigen Receptor and Interleukin-15. Clin Cancer Res (2019) 25:2915–24. doi: 10.1158/1078-0432.CCR-18-1811 30617136

[26] Lanitis E Rota G Kosti P Ronet C Spill A Seijo B. Optimized Gene Engineering of Murine CAR-T Cells Reveals the Beneficial Effects of IL-15 Coexpression. J Exp Med (2021) 218:1–19. doi: 10.1084/jem.20192203 PMC7653685 33156338

[27] Tang N Cheng C Zhang X Qiao M Li N Mu W. TGF-β Inhibition *via* CRISPR Promotes the Long-Term Efficacy of CAR T Cells Against Solid Tumors. JCI Insight (2020) 5:1–17. doi: 10.1172/jci.insight.133977 PMC7101140 31999649

[28] Shum T Omer B Tashiro H Kruse RL Wagner DL Parikh K. Constitutive Signaling From an Engineered IL7 Receptor Promotes Durable Tumor Elimination by Tumor-Redirected T Cells. Cancer Discovery (2017) 7:1238–47. doi: 10.1158/2159-8290.CD-17-0538 PMC5669830 28830878

[29] Mohammed S Sukumaran S Bajgain P Watanabe N Heslop HE Rooney CM. Improving Chimeric Antigen Receptor-Modified T Cell Function by Reversing the Immunosuppressive Tumor Microenvironment of Pancreatic Cancer. Mol Ther (2017) 25:249–58. doi: 10.1016/j.ymthe.2016.10.016 PMC5363304 28129119

[30] Weimin S Abula A Qianghong D Wenguang W. Chimeric Cytokine Receptor Enhancing PSMA-CAR-T Cell-Mediated Prostate Cancer Regression. Cancer Biol Ther (2020) 21:570–80. doi: 10.1080/15384047.2020.1739952 PMC7515537 32208880

[31] Lange S Sand LG Bell M Patil SL Langfitt D Gottschalk S. A Chimeric GM-CSF/IL18 Receptor to Sustain CAR T-Cell Function. Cancer Discovery (2021) 7:1661–71. doi: 10.1158/2159-8290.CD-20-0896 PMC8292158 33563660

[32] Chen X Yang S Li S Qu Y Wang HY Liu J. Secretion of Bispecific Protein of Anti-PD-1 Fused With TGF-β Trap Enhances Antitumor Efficacy of CAR-T Cell Therapy. Mol Ther Oncol (2021) 21:144–57. doi: 10.1016/j.omto.2021.03.014 PMC8082048 33981830

[33] Luo H Su J Sun R Sun Y Wang Y Dong Y. Coexpression of IL7 and CCL21 Increases Efficacy of CAR-T Cells in Solid Tumors Without Requiring Preconditioned Lymphodepletion. Clin Cancer Res (2020) 26:5494–505. doi: 10.1158/1078-0432.CCR-20-0777 32816947

[34] Hurton LV Singh H Najjar AM Switzer KC Mi T Maiti S. Tethered IL-15 Augments Antitumor Activity and Promotes a Stem-Cell Memory Subset in Tumor-Specific T Cells. Proc Natl Acad Sci (2016) 113:E7788–97. doi: 10.1073/pnas.1610544113 PMC5137758 27849617

[35] Jin L Cao L Zhu Y Cao J Li X Zhou J. Enhance Anti-Lung Tumor Efficacy of Chimeric Antigen Receptor-T Cells by Ectopic Expression of C–C Motif Chemokine Receptor 6. Sci Bull (2020) 8:803–12. doi: 10.1016/j.scib.2020.12.027 36654137

[36] Craddock JA Lu A Bear A Pule M Brenner MK Rooney CM. Enhanced Tumor Trafficking of GD2 Chimeric Antigen Receptor T Cells by Expression of the Chemokine Receptor CCR2b. J Immunother (Hagerstown Md. 1997) (2010) 33:780–8. doi: 10.1097/CJI.0b013e3181ee6675 PMC2998197 20842059

[37] Di Stasi A De Angelis B Rooney CM Zhang L Mahendravada A Foster AE. T Lymphocytes Coexpressing CCR4 and a Chimeric Antigen Receptor Targeting CD30 Have Improved Homing and Antitumor Activity in a Hodgkin Tumor Model. Blood (2009) 113:6392–402. doi: 10.1182/blood-2009-03-209650 PMC2710932 19377047

[38] Liu G Rui W Zheng H Huang D Yu F Zhang Y. CXCR2-Modified CAR-T Cells Have Enhanced Trafficking Ability That Improves Treatment of Hepatocellular Carcinoma. Eur J Immunol (2020) 50:712–24. doi: 10.1002/eji.201948457 31981231

[39] Kang L Tang X Xu N Li M Tan J Qi W. shRNA-Interleukin-6 Modified CD19-Specific Chimeric Antigen Receptor T Cell Significantly Improves the Safety in Acute Lymphoblastic Leukemia. Blood (2019) 134:2621–1. doi: 10.1182/blood-2019-132067

[40] Tan AHJ Vinanica N Campana D. Chimeric Antigen Receptor-T Cells With Cytokine Neutralizing Capacity. Blood Adv (2020) 4:1419–31. doi: 10.1182/bloodadvances.2019001287 PMC7160280 32271901

[41] Sterner RM Sakemura R Cox MJ Yang N Khadka RH Forsman CL. GM-CSF Inhibition Reduces Cytokine Release Syndrome and Neuroinflammation But Enhances CAR-T Cell Function in Xenografts. Blood (2019) 133:697–709. doi: 10.1182/blood-2018-10-881722 30463995 PMC6376281

[42] Avanzi MP Yeku O Li X Wijewarnasuriya DP van Leeuwen DG Cheung K. Engineered Tumor-Targeted T Cells Mediate Enhanced Anti-Tumor Efficacy Both Directly and Through Activation of the Endogenous Immune System. Cell Rep (2018) 23:2130–41. doi: 10.1016/j.celrep.2018.04.051 PMC5986286 29768210

[43] Pegram HJ Lee JC Hayman EG Imperato GH Tedder TF Sadelain M. Tumor-Targeted T Cells Modified to Secrete IL-12 Eradicate Systemic Tumors Without Need for Prior Conditioning. Blood (2012) 119:4133–41. doi: 10.1182/blood-2011-12-400044 PMC3359735 22354001

[44] Koneru M Purdon TJ Spriggs D Koneru S Brentjens RJ. IL-12 Secreting Tumor-Targeted Chimeric Antigen Receptor T Cells Eradicate Ovarian Tumors *In Vivo* . Oncoimmunology (2015) 4:e994446. doi: 10.4161/2162402X.2014.994446 25949921 PMC4404840

[45] Li X Daniyan AF Lopez AV Purdon TJ Brentjens RJ. Cytokine IL-36γ Improves CAR T-Cell Functionality and Induces Endogenous Antitumor Response. Leukemia (2021) 35:506–21. doi: 10.1038/s41375-020-0874-1 PMC7680719 32447345

[46] Singh H Figliola MJ Dawson MJ Olivares S Zhang L Yang G. Manufacture of Clinical-Grade CD19-Specific T Cells Stably Expressing Chimeric Antigen Receptor Using Sleeping Beauty System and Artificial Antigen Presenting Cells. PLoS One (2013) 8:e64138. doi: 10.1371/journal.pone.0064138 23741305 PMC3669363

[47] Louis CU Savoldo B Dotti G Pule M Yvon E Myers GD. Antitumor Activity and Long-Term Fate of Chimeric Antigen Receptor–Positive T Cells in Patients With Neuroblastoma. Blood J Am Soc Hematol (2011) 118:6050–6. doi: 10.1182/blood-2011-05-354449 PMC3234664 21984804

[48] Maude SL Frey N Shaw PA Aplenc R Barrett DM Bunin NJ. Chimeric Antigen Receptor T Cells for Sustained Remissions in Leukemia. N Engl J Med (2014) 371:1507–17. doi: 10.1056/NEJMoa1407222 PMC4267531 25317870

[49] Jafarzadeh L Masoumi E Fallah-Mehrjardi K Mirzaei HR Hadjati J. Prolonged Persistence of Chimeric Antigen Receptor (CAR) T Cell in Adoptive Cancer Immunotherapy: Challenges and Ways Forward. Front Immunol (2020) 11:702. doi: 10.3389/fimmu.2020.00702 32391013 PMC7188834

[50] Stock S Schmitt M Sellner L. Optimizing Manufacturing Protocols of Chimeric Antigen Receptor T Cells for Improved Anticancer Immunotherapy. Int J Mol Sci (2019) 20:1–21. doi: 10.3390/ijms20246223 PMC6940894 31835562

[51] McLellan AD Ali Hosseini Rad SM. Chimeric Antigen Receptor T Cell Persistence and Memory Cell Formation. Immunol Cell Biol (2019) 97:664–74. doi: 10.1111/imcb.12254 31009109

[52] Liu L Bi E Ma X Xiong W Qian J Ye L. Enhanced CAR-T Activity Against Established Tumors by Polarizing Human T Cells to Secrete Interleukin-9. Nat Commun (2020) 11:5902. doi: 10.1038/s41467-020-19672-2 33214555 PMC7677397

[53] Kagoya Y Tanaka S Guo T Anczurowski M Wang CH Saso K. A Novel Chimeric Antigen Receptor Containing a JAK-STAT Signaling Domain Mediates Superior Antitumor Effects. Nat Med (2018) 24:352–9. doi: 10.1038/nm.4478 PMC5839992 29400710

[54] Siegel AM Heimall J Freeman AF Hsu AP Brittain E Brenchley JM. A Critical Role for STAT3 Transcription Factor Signaling in the Development and Maintenance of Human T Cell Memory. Immunity (2011) 35:806–18. doi: 10.1016/j.immuni.2011.09.016 PMC3228524 22118528

[55] Cui W Liu Y Weinstein JS Craft J Kaech SM. An Interleukin-21-Interleukin-10-STAT3 Pathway is Critical for Functional Maturation of Memory CD8+ T Cells. Immunity (2011) 35:792–805. doi: 10.1016/j.immuni.2011.09.017 22118527 PMC3431922

[56] Sabatino M Hu J Sommariva M Gautam S Fellowes V Hocker JD. Generation of Clinical-Grade CD19-Specific CAR-Modified CD8+ Memory Stem Cells for the Treatment of Human B-Cell Malignancies. Blood (2016) 128:519–28. doi: 10.1182/blood-2015-11-683847 PMC4965906 27226436

[57] Cieri N Camisa B Cocchiarella F Forcato M Oliveira G Provasi E. IL-7 and IL-15 Instruct the Generation of Human Memory Stem T Cells From Naive Precursors. Blood (2013) 121:573–84. doi: 10.1182/blood-2012-05-431718 23160470

[58] Ghosh A Smith M James SE Davila ML Velardi E Argyropoulos KV. Donor CD19 CAR T Cells Exert Potent Graft-*Versus*-Lymphoma Activity With Diminished Graft-*Versus*-Host Activity. Nat Med (2017) 23:242–9. doi: 10.1038/nm.4258 PMC5528161 28067900

[59] Turley SJ Cremasco V Astarita JL. Immunological Hallmarks of Stromal Cells in the Tumour Microenvironment, Nature Reviews. Immunology (2015) 15:669–82. doi: 10.1038/nri3902 26471778

[60] Principe DR Doll JA Bauer J Jung B Munshi HG Bartholin L. TGF-β: Duality of Function Between Tumor Prevention and Carcinogenesis. J Natl Cancer Inst (2014) 106:djt369. doi: 10.1093/jnci/djt369 24511106 PMC3952197

[61] Ramesh S Wildey GM Howe PH. Transforming Growth Factor Beta (TGFbeta)-Induced Apoptosis: The Rise & Fall of Bim. Cell Cycle (Georgetown Tex.) (2009) 8:11–7. doi: 10.4161/cc.8.1.7291 PMC3191464 19106608

[62] Weller M Constam DB Malipiero U Fontana A. Transforming Growth Factor-Beta 2 Induces Apoptosis of Murine T Cell Clones Without Down-Regulating Bcl-2 mRNA Expression. Eur J Immunol (1994) 24:1293–300. doi: 10.1002/eji.1830240608 8206089

[63] Belli C Trapani D Viale G D'Amico P Duso BA Della Vigna P. Targeting the Microenvironment in Solid Tumors. Cancer Treat Rev (2018) 65:22–32. doi: 10.1016/j.ctrv.2018.02.004 29502037

[64] Wherry EJ Kurachi M. Molecular and Cellular Insights Into T Cell Exhaustion. Nat Rev Immunol (2015) 15:486–99. doi: 10.1038/nri3862 PMC4889009 26205583

[65] Davoodzadeh Gholami M Kardar GA Saeedi Y Heydari S Garssen J Falak R. Exhaustion of T Lymphocytes in the Tumor Microenvironment: Significance and Effective Mechanisms. Cell Immunol (2017) 322:1–14. doi: 10.1016/j.cellimm.2017.10.002 29079339

[66] Jiang Y Li Y Zhu B. T-Cell Exhaustion in the Tumor Microenvironment. Cell Death Dis (2015) 6:e1792. doi: 10.1038/cddis.2015.162 26086965 PMC4669840

[67] Pellegrini M Calzascia T Elford AR Shahinian A Lin AE Dissanayake D. Adjuvant IL-7 Antagonizes Multiple Cellular and Molecular Inhibitory Networks to Enhance Immunotherapies. Nat Med (2009) 15:528–36. doi: 10.1038/nm.1953 19396174

[68] Wu T Ji Y Moseman EA Xu HC Manglani M Kirby M. The TCF1-Bcl6 Axis Counteracts Type I Interferon to Repress Exhaustion and Maintain T Cell Stemness. Sci Immunol (2016) 1:1–27. doi: 10.1126/sciimmunol.aai8593 PMC5179228 28018990

[69] Chen Z Ji Z Ngiow SF Manne S Cai Z Huang AC. TCF-1-Centered Transcriptional Network Drives an Effector *Versus* Exhausted CD8 T Cell-Fate Decision. Immunity (2019) 51:840–855.e845. doi: 10.1016/j.immuni.2019.09.013 31606264 PMC6943829

[70] Alizadeh D Wong RA Yang X Wang D Pecoraro JR Kuo C-F. IL-15-Mediated Reduction of Mtorc1 Activity Preserves the Stem Cell Memory Phenotype of CAR-T Cells and Confers Superior Antitumor Activity. Cancer Immunol Res (2019) 5:759–72. doi: 10.1158/2326-6066.CIR-18-0466 PMC6687561 30890531

[71] Giuffrida L Sek K Henderson MA House IG Lai J Chen AX. IL-15 Preconditioning Augments CAR T Cell Responses to Checkpoint Blockade for Improved Treatment of Solid Tumors. Mol Ther (2020) 28:2379–93. doi: 10.1016/j.ymthe.2020.07.018 PMC7647667 32735774

[72] Narayan V Barber-Rotenberg J Fraietta J Hwang W-T Lacey SF Plesa G. A Phase I Clinical Trial of PSMA-Directed/Tgfβ-Insensitive CAR-T Cells in Metastatic Castration-Resistant Prostate Cancer. J Clin Oncol (2021) 39:125–5. doi: 10.1200/JCO.2021.39.6_suppl.125

[73] Green DR Droin N Pinkoski M. Activation-Induced Cell Death in T Cells. Immunol Rev (2003) 193:70–81. doi: 10.1034/j.1600-065X.2003.00051.x 12752672

[74] Otano I Alvarez M Minute L Ochoa MC Migueliz I Molina C. Human CD8 T Cells are Susceptible to TNF-Mediated Activation-Induced Cell Death. Theranostics (2020) 10:4481–9. doi: 10.7150/thno.41646 PMC7150490 32292509

[75] Marks-Konczalik J Dubois S Losi JM Sabzevari H Yamada N Feigenbaum L. IL-2-Induced Activation-Induced Cell Death is Inhibited in IL-15 Transgenic Mice. Proc Natl Acad Sci USA (2000) 97:11445–50. doi: 10.1073/pnas.200363097 PMC17219 11016962

[76] Arakaki R Yamada A Kudo Y Hayashi Y Ishimaru N. Mechanism of Activation-Induced Cell Death of T Cells and Regulation of FasL Expression. Crit Rev Immunol (2014) 34:301–14. doi: 10.1615/CritRevImmunol.2014009988 24941158

[77] Brown CE Vishwanath RP Aguilar B Starr R Najbauer J Aboody KS. Tumor-Derived Chemokine MCP-1/CCL2 is Sufficient for Mediating Tumor Tropism of Adoptively Transferred T Cells. J Immunol (2007) 179:3332–41. doi: 10.4049/jimmunol.179.5.3332 17709550

[78] Galon J Costes A Sanchez-Cabo F Kirilovsky A Mlecnik B Lagorce-Pagès C. Type, Density, and Location of Immune Cells Within Human Colorectal Tumors Predict Clinical Outcome. Science (2006) 313:1960–4. doi: 10.1126/science.1129139 17008531

[79] Kmiecik J Poli A Brons NH Waha A Eide GE Enger PØ. Elevated CD3+ and CD8+ Tumor-Infiltrating Immune Cells Correlate With Prolonged Survival in Glioblastoma Patients Despite Integrated Immunosuppressive Mechanisms in the Tumor Microenvironment and at the Systemic Level. J Neuroimmunol (2013) 264:71–83. doi: 10.1016/j.jneuroim.2013.08.013 24045166

[80] Ma S Li X Wang X Cheng L Li Z Zhang C. Current Progress in CAR-T Cell Therapy for Solid Tumors. Int J Biol Sci (2019) 15:2548–60. doi: 10.7150/ijbs.34213 PMC6854376 31754328

[81] Martinez M Moon EK. CAR T Cells for Solid Tumors: New Strategies for Finding, Infiltrating, and Surviving in the Tumor Microenvironment. Front Immunol (2019) 10. doi: 10.3389/fimmu.2019.00128 PMC6370640 30804938

[82] Caruana I Savoldo B Hoyos V Weber G Liu H Kim ES. Heparanase Promotes Tumor Infiltration and Antitumor Activity of CAR-Redirected T Lymphocytes. Nat Med (2015) 21:524–9. doi: 10.1038/nm.3833 PMC4425589 25849134

[83] Wang L-CS Lo A Scholler J Sun J Majumdar RS Kapoor V. Targeting Fibroblast Activation Protein in Tumor Stroma With Chimeric Antigen Receptor T Cells can Inhibit Tumor Growth and Augment Host Immunity Without Severe Toxicity. Cancer Immunol Res (2014) 2:154–66. doi: 10.1158/2326-6066.CIR-13-0027 PMC4007316 24778279

[84] Kanagawa N Yanagawa T Nakagawa T Okada N Nakagawa S. Tumor Vessel-Injuring Ability Improves Antitumor Effect of Cytotoxic T Lymphocytes in Adoptive Immunotherapy. Cancer Gene Ther (2013) 20:57–64. doi: 10.1038/cgt.2012.85 23175243 PMC3534155

[85] Maude SL Barrett D Teachey DT Grupp SA. Managing Cytokine Release Syndrome Associated With Novel T Cell-Engaging Therapies. Cancer J (Sudbury Mass.) (2014) 20:119. doi: 10.1097/PPO.0000000000000035 PMC4119809 24667956

[86] Chen H Wang F Zhang P Zhang Y Chen Y Fan X. Management of Cytokine Release Syndrome Related to CAR-T Cell Therapy. Front Med (2019) 13:610–7. doi: 10.1007/s11684-019-0714-8 31571160

[87] Wei J Liu Y Wang C Zhang Y Tong C Dai G. The Model of Cytokine Release Syndrome in CAR T-Cell Treatment for B-Cell non-Hodgkin Lymphoma. Signal Transduct Targeted Ther (2020) 5:134. doi: 10.1038/s41392-020-00256-x PMC7388484 32728035

[88] Lee DW Kochenderfer JN Stetler-Stevenson M Cui YK Delbrook C Feldman SA. T Cells Expressing CD19 Chimeric Antigen Receptors for Acute Lymphoblastic Leukaemia in Children and Young Adults: A Phase 1 Dose-Escalation Trial. Lancet (Lond Engl) (2015) 385:517–28. doi: 10.1016/S0140-6736(14)61403-3 PMC7065359 25319501

[89] Wang Y Zhang WY Han QW Liu Y Dai HR Guo YL. Effective Response and Delayed Toxicities of Refractory Advanced Diffuse Large B-Cell Lymphoma Treated by CD20-Directed Chimeric Antigen Receptor-Modified T Cells. Clin Immunol (Orlando Fla.) (2014) 155:160–75. doi: 10.1016/j.clim.2014.10.002 25444722

[90] Fried S Avigdor A Bielorai B Meir A Besser MJ Schachter J. Early and Late Hematologic Toxicity Following CD19 CAR-T Cells. Bone Marrow Transplant (2019) 54:1643–50. doi: 10.1038/s41409-019-0487-3 30809033

[91] Bonifant CL Jackson HJ Brentjens RJ Curran KJ. Toxicity and Management in CAR T-Cell Therapy. Mol Ther Oncol (2016) 3:16011. doi: 10.1038/mto.2016.11 PMC5008265 27626062

[92] Kang L Tang X Zhang J Li M Xu N Qi W. Interleukin-6-Knockdown of Chimeric Antigen Receptor-Modified T Cells Significantly Reduces IL-6 Release From Monocytes. Exp Hematol Oncol (2020) 9:11. doi: 10.1186/s40164-020-00166-2 32523801 PMC7278071

[93] Sachdeva M Aranda-Orgilles B Duchateau P Poirot L Valton J. Abstract A60: GM-CSF Modulation Restricts the Secretion of Main Cytokines Associated With CAR T-Cell Induced Cytokine Release Syndrome. Cancer Immunol Res (2020) 8:A60–0. doi: 10.1158/2326-6074.TUMIMM19-A60

[94] Norelli M Camisa B Barbiera G Falcone L Purevdorj A Genua M. Monocyte-Derived IL-1 and IL-6 are Differentially Required for Cytokine-Release Syndrome and Neurotoxicity Due to CAR T Cells. Nat Med (2018) 24:739–48. doi: 10.1038/s41591-018-0036-4 29808007

[95] Taraseviciute A Tkachev V Ponce R Turtle CJ Snyder JM Liggitt HD. Chimeric Antigen Receptor T Cell-Mediated Neurotoxicity in Nonhuman Primates. Cancer Discov (2018) 8:750–63. doi: 10.1158/2159-8290.CD-17-1368 PMC6058704 29563103

[96] Gust J Hay KA Hanafi LA Li D Myerson D Gonzalez-Cuyar LF. Endothelial Activation and Blood-Brain Barrier Disruption in Neurotoxicity After Adoptive Immunotherapy With CD19 CAR-T Cells. Cancer Discov (2017) 7:1404–19. doi: 10.1158/2159-8290.CD-17-0698 PMC5718945 29025771

[97] Neelapu SS Locke FL Bartlett NL Lekakis LJ Miklos DB Jacobson CA. Axicabtagene Ciloleucel CAR T-Cell Therapy in Refractory Large B-Cell Lymphoma. N Engl J Med (2017) 377:2531–44. doi: 10.1056/NEJMoa1707447 PMC5882485 29226797

[98] Tanyi JL Stashwick C Plesa G Morgan MA Porter D Maus MV. Possible Compartmental Cytokine Release Syndrome in a Patient With Recurrent Ovarian Cancer After Treatment With Mesothelin-Targeted CAR-T Cells. J Immunother (Hagerstown Md. 1997) (2017) 40:104–7. doi: 10.1097/CJI.0000000000000160 28234665

[99] Brossart P. The Role of Antigen Spreading in the Efficacy of Immunotherapies. Clin Cancer Res (2020) 26:4442–7. doi: 10.1158/1078-0432.CCR-20-0305 32357962

[100] Schneider D Xiong Y. Trispecific CD19-CD20-CD22-Targeting duoCAR-T Cells Eliminate Antigen-Heterogeneous B Cell Tumors in Preclinical Models. Sci Transl Med (2021) 13:586. doi: 10.1126/scitranslmed.abc6401 33762438

[101] Alizadeh D Wong RA Gholamin S Maker M Aftabizadeh M Yang X. IFNg Is Critical for CAR T Cell Mediated Myeloid Activation and Induction of Endogenous Immunity. Cancer Discov (2021) 9:2248–65. doi: 10.1158/2159-8290.CD-20-1661 PMC8561746 33837065

[102] Mirzaei HR Hadjati J. Commentary: IL-12-Secreting Tumor-Targeted Chimeric Antigen Receptor T Cells: An Unaddressed Concern on Koneru Et al. (2015). Oncoimmunology (2016) 5:e1100792. doi: 10.1080/2162402X.2015.1100792 27141362 PMC4839381

[103] Kueberuwa G Kalaitsidou M Cheadle E Hawkins RE Gilham DE. CD19 CAR T Cells Expressing IL-12 Eradicate Lymphoma in Fully Lymphoreplete Mice Through Induction of Host Immunity. Mol Ther Oncol (2018) 8:41–51. doi: 10.1016/j.omto.2017.12.003 PMC5772011 29367945

